# Description of *Culicoides truuskae* sp. n. (Diptera: Ceratopogonidae) from southern Africa

**DOI:** 10.4102/ojvr.v90i1.2072

**Published:** 2023-03-06

**Authors:** Karien Labuschagne, Rudolf Meiswinkel, Danica Liebenberg, Carissa van Zyl, Antoinette van Schalkwyk, Clarke Scholtz

**Affiliations:** 1 Department of Entomology, Faculty of Epidemiology, Parasites and Vectors, Agricultural Research Council – Onderstepoort Veterinary Research, Onderstepoort, South Africa; 2 Department of Zoology and Entomology, Faculty of Natural and Agricultural Sciences, University of Pretoria, Pretoria, South Africa; 3 Private, Rome, Italy; 4 Unit for Environmental Sciences and Management, Faculty of Natural and Agricultural Science, North-West University, Potchefstroom, South Africa; 5 Vaccines and Diagnostics Development Programme, Agricultural Research Council – Onderstepoort Veterinary Research, Onderstepoort, South Africa

**Keywords:** *Culicoides*, taxonomy, description new species, DNA barcoding, map

## Abstract

*Culicoides truuskae* Labuschagne and Meiswinkel sp. n. is described and illustrated in both sexes from material collected in South Africa and Namibia. It is restricted to the xeric western margin of the subcontinent, occurring in Fynbos, Nama-Karoo and Succulent Karoo ecoregions in South Africa and Desert and Savanna ecoregions in Namibia experiencing < 600 mm of rainfall annually. *Culicoides truuskae* sp. n. is part of the Afrotropical ‘plain-wing’ *Culicoides* in which the wing lacks a distinguishing pattern of light and dark spots; the diagnostic dark smudge that traverses wing cell r3 may result in *C. truuskae* sp. n. being misidentified as the sympatric but phyletically unrelated *Culicoides herero* (Enderlein) – (of the Similis group, subgenus Oecacta Poey). Additionally, this study is the first description of the male of *C. herero. C. truuskae* sp. n. and *Culicoides coarctatus* Clastrier and Wirth share similar characters in the male genitalia, although the two species are separable on wing pattern and female flagellum sensilla coeloconica (SCo) distribution. The breeding habitat and adult female blood-feeding preferences of C. *truuskae* sp. n. are not known. A maximum likelihood phylogenetic tree, using mitochondrial *cytochrome c oxidase* I (COI) sequence data, is provided to further clarify the relationship between *C. truuskae* sp. n., *C. coarctatus* and *C. herero*. Extensive light trap data, collected over 30 years, are used to map the distribution ranges of *C. truuskae* sp. n., *C. coarctatus* and *C. herero* in Southern Africa.

**Contribution:**

The description of this new species and the description of the male of *C. herero* increases our understanding of the diversity and distribution of *Culicoides* species in southern Africa.

## Introduction

In the genus *Culicoides* Latreille, the adult females of nearly all of the world’s approximately 1350 species are blood feeders; as a result, many species transmit viruses, protozoans and filarial nematodes to an array of vertebrates including humans and their domesticated livestock and wildlife (Borkent & Dominiak 2020; Meiswinkel, Venter & Nevill 2004; Mellor, Boorman & Baylis 2000). A number of the viruses transmitted by *Culicoides* are of veterinary significance, particularly in Africa, where the most important are the causative agents of African horse sickness, equine encephalosis and bluetongue of sheep.

The *Culicoides* wing bears a ‘… characteristic pattern of dark and light spots … and is of primary importance in classification; however, in some groups or species it is poorly developed or even absent’ (Blanton & Wirth 1979). As a rule, the wings of *Culicoides* from the forested tropics are far more brightly patterned (picture-wing) than the more dully coloured ones (plain-wing and half-wing) that predominate in drier and more open habitats. Of the 160 species that comprise the Afrotropical checklist, 17 are ‘plain-wing’ species, while an almost equal number possess a pattern that is ‘poorly developed’ and restricted to the basal half of the wing; these are referred to collectively as the ‘half-wing’ group, while the remainder possess a distinct pattern (Meiswinkel et al. 2004). As a result, more than 20% of Africa’s biting midge species are difficult to identify accurately based on wing pattern alone; accordingly, these species are slide-mounted in order to examine other distinguishing features at higher magnification (Meiswinkel 1995).

Wing pattern reduction or its total loss is more pronounced in the xeric ecoregions of the world; for example, more than one-third of the 34 species of *Culicoides* recorded from the Arabian Peninsula (Boorman 1989) comprise ‘plain-wing’ or ‘half-wing’ species. Globally, deserts form the core of many of the world’s drier tracts, with characteristic unstable, highly labile entities that expand and contract as climatic conditions vary. In Africa’s past during drier epochs, ‘arid corridors’ developed and expanded, cutting diagonally across the waist of the continent. This facilitated faunal and floral interchange between the deserts and xeric shrublands of the two ‘corners’ of the continent, namely the Horn of Africa and the Karoo-Namib. In mammals, the best examples of the existence of this ancient corridor are the oryx, dik-dik and bat-eared fox, while in birds it is among the bustards, larks, and smaller hornbills (Kingdon 1990). This interchange also likely involved insects. These past events complicate taxonomic studies because sister taxa, separated by vast distances and across timeframes of unknown length, may be common.

As observed, the taxonomy of the *Culicoides* of the Arabian Peninsula has enjoyed some attention (Boorman 1989), in contrast to the *Culcoides* species 5000 km away in the southwestern corner of Africa. This region is characterised by deserts and xeric shrublands that have long prevailed on the broad strip of land that runs for almost 2000 km along the Atlantic seaboard, an area of great dryness the early Khoisan peoples called ‘Karoo’. Phytogeographers have parceled this ‘great dryness’ into a number of terrestrial ecoregions; the one from which nearly all collections of *Culicoides truuskae* sp. n. were made is the ‘Succulent Karoo’ (ecoregion 110; Burgess et al. 2006) and recognised to be the ‘… world’s only plant hotspot that is entirely arid’ (Burgess et al. 2006). Explosive speciation among succulents and geophytes within the larger Karoo-Namib phytochorion (Van Wyk & Smith 2001) likely accounts for the co-existence of a rich complement of arthropod endemics, especially among the arachnids, hopliniid beetles, aculeate Hymenoptera, and reptiles (Burgess et al. 2006). Hopliniid monkey beetles, masarine wasps and colletid, fideliid, and melittid bees serve as important pollinators (Burgess et al. 2006). In sharp contrast, the bird (one strict endemic) and mammal faunas (three endemics, of which two are subterranean) are depauperate (Burgess et al. 2006).

Reduced vertebrate diversity will impinge greatly upon the feeding opportunities available to blood sucking insects such as *Culicoides.* However, it is equally valid to argue that sustained aridity, across evolutionary time, is strongly selective and so bound to have given rise to a specialised, albeit greatly reduced, biting midge fauna within the arid heart of southwestern Africa. This possibility remains unexplored.

In this study, the authors describe *C. truuskae* sp. n. from the western deserts and xeric shrublands of South Africa and Namibia. In addition, the male of *Culicoides herero* is described for the first time. At the species level, *C. truuskae* sp. n. is compared taxonomically with *Culicoides coarctatus* (because of shared characters of the male genitalia) and to *C. herero* (with which the new species may be confused owing to wing pattern similarity). The authors assess the higher taxonomic position of the three. Finally, extensive light trap data collected over 30 years allowed the authors to map in detail the distribution of the three species within southern Africa and to provide some data on the seasonal phenology of its constituent species.

## Material and methods

All specimens of *C. truuskae* sp. n. examined were collected with the Onderstepoort Veterinary Institute (OVI) black light suction trap. A small portion of this material is slide-mounted in Canada balsam and housed in the Insect collection of the Agricultural Research Council – Onderstepoort Veterinary Institute (ARC–OVI). Specimens were examined using a Zeiss Axiostar compound microscope equipped with an ocular reticule for measurements. All measurements are in micrometres (µm), except wing lengths (mm). Photographs were taken using a Zeiss AxioCam ERc5s camera, measurements were carried out in ZEN 2.3 lite and the images then enhanced in Adobe Photoshop (elements 3.0). In general, the descriptive taxonomic terminology follows Khamala and Kettle (1971) and Boorman (1989), except for (1) the flagellar segments numbered 1–13 (instead of referring to antennal segments 3–15) and (2) the proboscis/head (P/H) ratio is used instead of the inverse (H/P). P is the distance from the tip of the proboscis to the tormae and H the distance from the tormae to the insertion alveolus of the interocular seta. Terms for the various types of sensilla situated on the flagellum follow Meiswinkel (1995).

Total deoxyribonucleic acid (DNA) extracts were obtained from individual *Culicoides* using the ZR Tissue and Insect DNA Miniprep kit (Zymo Research) and following the manufacturer’s instructions. The wings, head and antennae were dissected off and mounted onto a microscope slide as a reference sample for each specimen before destructive DNA extraction was performed for female specimens. For male specimens, the wings and the genitalia were dissected of and mounted before extraction. A 700 base pairs (bp) region of the mitochondrial *cytochrome c oxidase* I (COI) gene amplified using the primers LC01490: GGTCAACAAATCATAAAGATATTGG and HC02198: TAAACTTCAGGGTGACCAAAAAATCA as previously described (Folmer et al. 1994).

Individual reactions were performed using Terra polymerase chain reaction (PCR) direct polymerase mix (Takara, Clontech) at an annealing temperature of 55 °C following manufacturer’s instructions. Amplicons sent for sequencing to Inqaba Biotech (Pretoria, South Africa), using the same PCR primers, in BigDye Terminator version 3.1 cycle sequencing reactions (Applied Biosystems, Foster City, California, United States) and analysed on an ABI3500-XL Genetic analyser.

Consensus sequences comprising individual COI gene fragments were compiled by assembling the forward and reverse sequences in CLC Genomics Workbench 8.0.1 (CLC Genomics Workbench 8.0.1, Qiagen Aarhus). New consensus sequences were deposited in GenBank (Accession numbers: KY305212, KY305213, KY305215–KY305221, KY305225, KY933263–KY933277, OP132687–OP132691) (Online Appendix 1 Table 1-A1). An alignment containing all COI consensus sequences, as well as selected reference sequences obtained from GenBank was constructed in Mega X using Clustal W (Tamura et al. 2013). The best fitting model was determined using Mega X, and a General Time Reversible model with Gamma distribution (4) and Invariant sites (G + I) was used to compute the Maximum Likelihood phylogeny with 1000 bootstrap replicates. Percentage sequence identity was determined between *C. truuskae* sp. n. sequences as well as between *C. truuskae* sp. n., *C. coarctatus* and *C. herero* and remaining sequences in the alignment using CLC Genomics Workbench 9.0.1. (www.CLC genomics.com).

The distribution map of *C. truuskae* sp. n, *C. coarctatus* and *C. herero* in southern Africa was generated using QGIS 22.7 software (QGIS Development Team 2022). The seasonal abundance and monthly prevalence of *C. truuskae* sp. n., *C. coarctatus* and *C. herero* is depicted in a single Mondrian Matrix; the matrix consists of squares colour-coded according to the log10 (*n* + 1) transformed monthly average abundance of each species.

### Ethical considerations

This article followed all ethical standards for research without direct contact with human or animal subjects.

## Results

### Taxonomy

**FIGURE 1:**
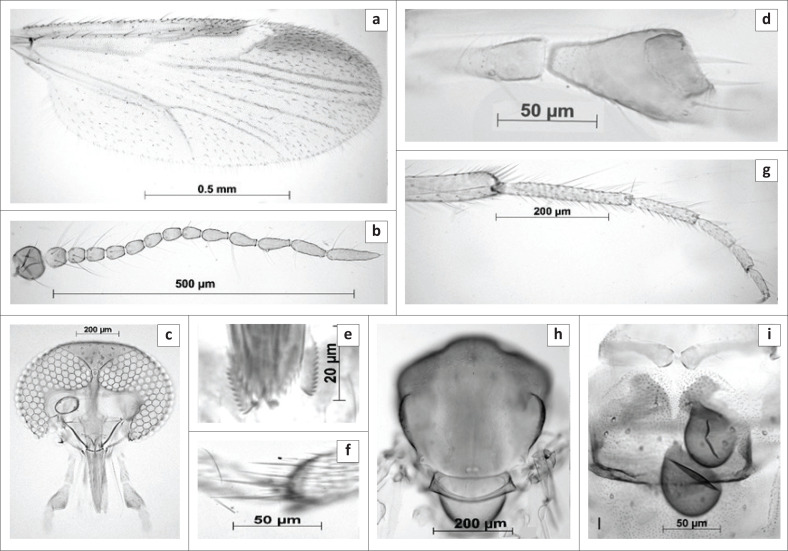
Female of *Culicoides truuskae* sp. n. (a) wing, (b) antenna, (c) head, (d) The third segment of maxillary palpus, (e) mandibular teeth, (f) tibial comb of hind leg, (g) hind leg, (h) thorax, (i) abdomen showing the spermathecae and the chitinous plates surrounding the gonopore.

**FIGURE 2:**
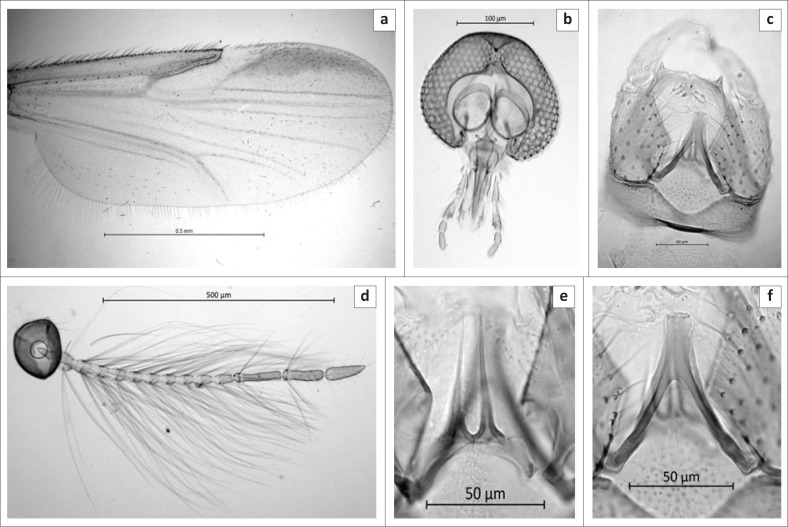
Male of *Culicoides truuskae* sp. n. (a) Wing, (b) Antenna, (c) Head, (d) Genitalia, (e) Parameres, (f) Aedeagus.

*Culicoides truuskae* Labuschagne and Meiswinkel, sp. n.

(Figure 1 and Figure 2).

*Type material* (84 females, 45 males).

Holotype female: South Africa: Northern Cape province; Augrabies Falls (-28.62, 20.36), 20/xi/1987, R Meiswinkel at livestock, ultraviolet (UV) light trap. Deposited in ARC-Onderstepoort Veterinary Institute, EPV, National Insect Collection, South Africa.

Paratypes: South Africa: Northern Cape province: Augrabies Falls (-28.62, 20.36), 20/ix/1987, R Meiswinkel, 7 females, 2 males; Springbok (-29.67, 17.88), 15/x/2009, 16/x/2008, 4/xi/2008, 18/xi/2008, 28/i/2009, 6/ii/2009, 16/ii/2009, 17/ii/2009, 6/iii/2009, 12/iii/2011, C Schutte, 65 females, 32 males; Upington (-28.66, 20.48), 24/x/2009, K Labuschagne, 2 females; South Africa: Western Cape Province: Piketberg (-32.84, 18.82), 10/i/2006, K Labuschagne, 1 female; Namibia: Aus (-26.66, 16.26), 8/2/13 D Liebenberg, 10 females, 11 males.

#### Differential diagnosis

Medium sized, fresh specimens brown and slide mounted specimens light brown, female eyes narrowly separated. Sensilla coeloconica (SCo) was present on flagellomeres 1, 3, 5, 7, 9–12 in females and 1, 11–12 in males. The third palpal segment inflated, sensory pit wide and deep. Wing was without pale or dark markings and anterior half of cell r_3_ was noticeably darkened. Spermathecae: two slightly unequal, ovoid spermathecae with a rudimentary third spermatheca present, sclerotised ring absent. Male genitalia: Ninth tergum with long, basally stout apirolateral processes; ninth sternum with broad and moderately deep caudomedian emargination, the ventral membrane spiculate; dorsal root of basistyle moderately short and stout, ventral root short; aedeagus triangular, narrowing gradually to broad apex, aedeagal arch moderately high; parameres broadly joined at the base mesally, each paramere slender, tapering to a single fine point.

#### Description

*Female (Figure 1): n* = 84.

*Head* (Figure 1c). Eyes bare, narrowly separated by distance of approximately one ocular facet. Flagellum (Figure 1b): Lengths of flagellomeres 1–13: 53/38/40/39/41/37/38/38/54/52/62/66/98 µm. Mean antennal ratio (AR): 1.08 (0.96–1.15). Mean number and distribution of SCo on flagellomeres 1–13: 3.01/0.05/0.98/0.02/0.98/0.03/1/0.01/1/1/1/2.86/0; range in number of SCo on flagellomeres 1–13: 2-5/0-1/0-1/0-1/1/0-1/1-2/0-1/1/1/1/1–4/0. Number and distribution of sensilla chaetica on flagellomeres 1–13: 7/7/7/6/8/6/7/7/2/2/2/2/5. Number and distribution of long and short blunt-tipped sensilla trichodea on flagellomeres 1–8: two long (LL) on flagellomere 1, and two long (LL) and one short (c) on flagellomeres 2–8. Palpus (Figure 1d) with lengths of segments 1 + 2, 3 − 5: 76/78/23/30. Mean palpal ratio (PR): 2.02 (1.95–2.05); the third segment strongly inflated with single, moderately deep and wide sensory pit. Mean P/H ratio: 0.81 (0.79–0). Mandible (Figure 1e) with 12-14 teeth.

*Thorax* (Figure 1h). Scutum light brown in slide mounted material with anterior promontories paler and dark brown in fresh specimens. No distinct pattern could be observed in ethanol preserved or slide mounted material. Halteres pale. Wing (Figure 1a): Mean length: 1.39 mm (1.11 mm – 1.6 mm). Mean costal ratio (CR): 0.61 (0.59–0.62). Mean wing length/width ratio: 2.21 (2.16–2.24). Wing without any markings visible, anterior half of cell r3 noticeably darker, macrotrichia dense. Macrotrichia moderately dense, sparse in radial cells and in r3 from next to the radial cells to the r-m crossvein; radial cells completely formed. Legs without pale bands (Figure 1g), fourth tarsomeres cylindrical; only the middle leg bearing a pair of short, erect spines apically on tarsomeres 1–4. Hind tibial comb with four spines (Figure 1f), second spine fractionally longer. Mean tarsal ratio (T/R): 1.83 (1.80–1.87).

*Abdomen*. Two, fully functional, ovoid spermathecae, each with slender, moderately short, narrow neck (Figure 1I) measuring 67.5 µm × 49.9 µm and 57.9 µm × 46.6 µm, third rudimentary spermatheca slender measuring 14.4 µm × 5.2 µm, without sclerotised ring near junction of ducts.

***Male* (Figure 2): *n* = 45.**

*Head* (Figure 2b). Eyes contiguous for less than half of an ocular facet. Flagellum (Figure 2d): Lengths of flagellomeres 1–13: 82/41/43/44/39/41/40/41/42/49/114/101/102. Mean antennal ratio (AR): 1.04 (0.96–1.10). Mean number and distribution of SCo on flagellomeres 1–13: 2/0/0/0/0/0/0/0/0/0/1.7/3.4/0; range in number of SCo on flagellomeres 1–13: 2–3/0/0/0/0/0/0/0/0/0/1–2/2–5/0. Number and distribution of sensilla chaetica on flagellomeres 11–13: 7/8/0. Number and distribution of long and short-tipped sensilla trichodea on flagellomeres 1–8: two long on flagellomere 1, two long and two short on flagellomeres 2–4 and one short on 5–9. Palpus (Figure 2b): Mean PR: 3.35 (3.09–3.61); the third segment narrow bearing single shallow sensory pit. Mean P/H: 0.54 (0.49–0.56).

*Thorax.* Scutum brown, anterior promontories paler. Wing (Figure 2a): Mean length: 1.14 mm (1.00 mm – 1.44 mm). Mean costal ratio (CR): 0.54 (0.51–0.56). Mean wing length/width ratio 2.62 (2.57–2.69).

*Abdomen.* Genitalia (Figure 2c, e–f) stout, square. Gonocoxite significantly broader basally, ventral root short; dorsal root stout, moderately long and straight, tip broadly rounded. Gonostyle basal two-third broadly inflated, narrowing quite abruptly to sharply pointed tips. Parameres broadly fused in mid-line, the distal portion extending posteriad, each paramere tapering into long, slender, slightly bowed, inwardly curved, fine, erect point (Figure 2f). Aedeagus robust, triangular, well-pigmented, narrowing gradually to broad apex, aedeagal arch moderately high, slightly convex, basal feet well-developed, projecting laterad at right angles (Figure 2e). Sternum 9 with broad and moderately deep caudomedian excavation; posterior membrane rather densely spiculate, spiculation extending posteriad over basal arch of aedeagus. Tergum 9 trapezoidal, narrowing gradually; apicolateral processes moderately broadly separated each process short, broad basally; caudal margin of tergum with small, indistinct notch medianally.

## Etymology

Named in honour of Dr Truuske Gerdes in recognition of her work on arboviral diseases of veterinary importance in South Africa.

## Bionomics

The immature stages, larval habitat, blood-feeding habits and vector status of *C. truuskae* sp. n. remain undescribed.

## Distribution

South Africa: Eastern, Northern and Western Cape provinces. Central and southern Namibia in: Erongo, Karas, Khomas and Otjozondjupa regions.

***Culicoides herero*** (Enderlein) 1908: 460 (*Ceratopogon*). Namibia

Specimens examined (37 females, 26 males):

- South Africa: Eastern Cape: Middelburg (-31.47, 25.03), 1/xii/1970, EM Nevill 2 females; Northern Cape: Augrabies Falls (-28.62, 20.36), 20/ix/1987, R Meiswinkel 5 females, 3 males; Bray (-25.46, 23.70), 21/i/1969, EM Nevill 1 male; Calvinia (-31.49, 19.8), 1/x/1997, R Meiswinkel 1 male; Springbok (-29.67, 17.88), 25/ii/2009, 16/x/2008, 18/xi/2008, C Schutte 4 females, 1 male; Western Cape: Beaufort West (-32.07, 22.84), 1/ii/2006, K Labuschagne 1 female; Lamberts Bay (-32.08, 18.34), 1/x/1997, G v Eeden 1 female; Namibia: Aus (-26.67, 16.26), 8/iii/2013, D Liebenberg 18 females, 15 males; Corona (-23.39, 16.16), 11/v/2010, E Bekker 1 female).

### Differential diagnosis

Small sized, fresh specimens dark and slide mounted specimens light brown. Female eyes narrowly separated. Sensilla coeloconica present on flagellomeres 1–8 in females and 1, 6, 8–10 in males. The third palpal segment inflated, sensory pit wide and deep. Wing with indistinct 1st and 2nd costal spots present and a faint pale spot in cell cua1. Costal vein between the first and second costal spots dark. Spermathecae: two slightly unequal, ovoid spermathecae with rudimentary a third spermatheca present, and a sclerotised ring present. Male genitalia: ventral roots of basistyle with anterior processes connected by a very narrow hyaline membrane; shoulders of aedeagus with a pair of posteriorly directed, pointed processes; distal median process long and stout with truncate apex. Paramere with a large, anterolaterally directed basal knob; main stem straight, stout, with a large subapical ventral lobe; distal portion recurved to a slender, pointed apex, and with 5–6 lateral fringing spines.

### Description

**FIGURE 3:**
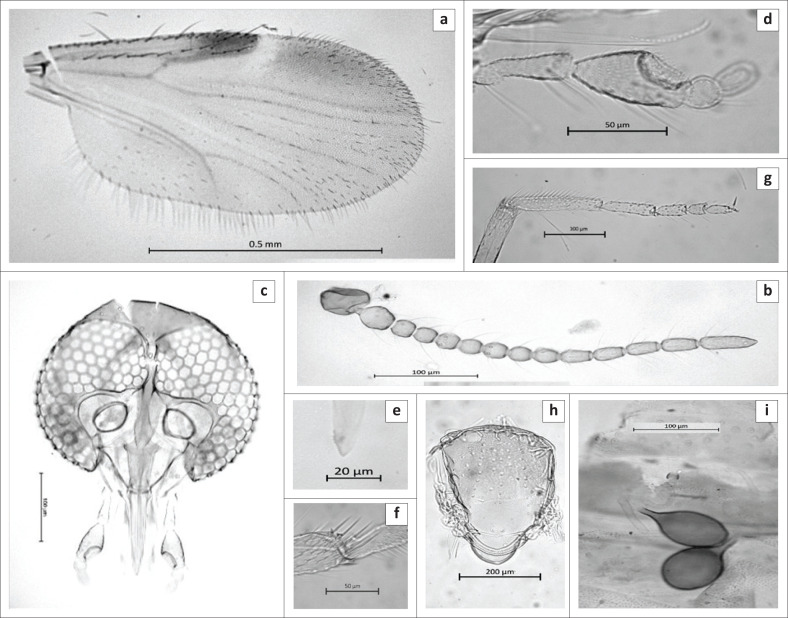
Female of *Culicoides herero.* (a) wing (female), (b) wing (male), (c) head (female), (d) abdomen showing the spermathecae and the chitinous plates surrounding the gonopore, (e) Male genitalia, (f) Aedeagus. (g) Paramere mandibular teeth, (h) antenna female, (i) antenna male.

Female (Figure 3): *n* = 37.

*Head* (Figure 3b). Eyes bare, narrowly separated by distance of approximately one ocular facet. Flagellum (Figure 3d):

- Lengths of flagellomeres 1–13: 41/23/22/23/24/24/22/26/30/29/31/36/54 µm. Mean antennal ratio (AR): 0.87 (0.85–0.89). Mean number and distribution of SCo on flagellomeres 1–13: 3.2/1/1/1/1/1.5/1/1.9/0/0/0/0/0; range in number of SCo on flagellomeres 1–13: 3–4/1/1/1/1/1–2/1/1/1–2/0/0/0/0. Number and distribution of sensilla chaetica on flagellomeres 1–13: 9/7/7/7/7/6/7/7/3/3/4/5/6. Number and distribution of long and short blunt-tipped sensilla trichodea on flagellomeres 1–13: two long (LL) on flagellomere 1 and two long (LL) and one short (c) on flagellomeres 2–8. Palpus (Figure 3d) with lengths of segments 1 + 2, 3 – 5: 56/62/22/25. Mean palpal ratio (PR): 2.01 (1.92–2.1); the third segment moderately to strongly inflated with a single, moderately deep and wide sensory pit. Mean P/H ratio: 0.60 (0.57–0.62). Mandible (Figure 3e) with 10–11 teeth.

*Thorax* (Figure 3h). Scutum light brown in slide mounted material and dark brown in fresh specimens. No distinct pattern could be observed in ethanol preserved or slide mounted material. Halter pale. Wing (Figure 3a): Mean length: 0.88 mm (0.77 mm – 1.05 mm). Mean costal ratio (CR): 0.53 (0.52–0.55). Mean wing length/width ratio: 2.06 (1.99–2.45). Wing pale, with indistinct first and second costal spots present, a faint pale spot in cell cua1, and anterior half of cell r3 noticeably darker. Macrotrichia moderately dense, absent only in radial cells and in r3 immediately below r1 and r2. Legs brown, tarsi paler, knees dark, tibiae with pale basal ring, only middle leg bearing a pair of short, erect spines apically on tarsomeres 1-4. Hind tibial comb with four spines, with the spine nearest the spur the longest. Mean tarsal ratio (T/R): 1.72 (1.56–1.80).

*Abdomen*: Two, fully functional, ovoid spermathecae, each with slender, moderately short, narrow neck (Figure 3e):

- measuring 55.1 µm × 39.5 µm and 38.1 µm × 34.1 µm, and a third rudimentary spermatheca slender measuring 19.6 µm × 3.8 µm, and a sclerotised ring present.

**FIGURE 4:**
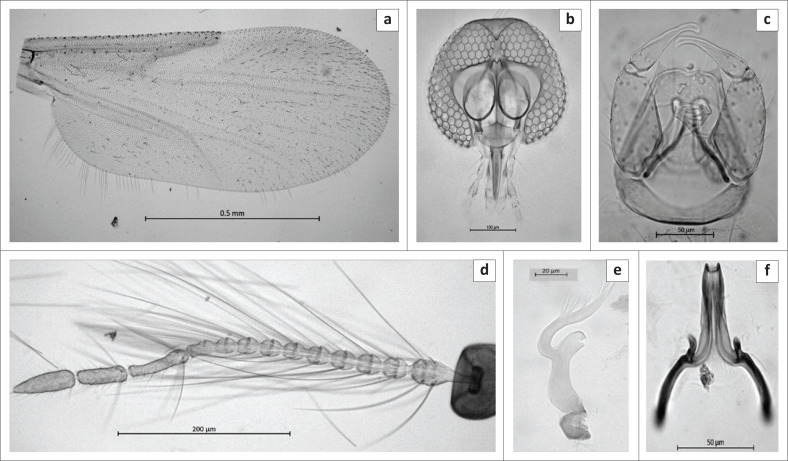
Male of *Culicoides herero* sp. n. (a) Wing, (b) Antenna, (c) Head, (d) Genitalia, (e) Parameres, (f) Aedeagus.

Male (Figure 4): *n* = 26.

*Head* (Figure 4b). Eyes separated by less than half width of an ommatidia. Flagellum (Figure 4d):

- Lengths of flagellomeres 1–13: 82/33/30/29/28/30/29/26/25/31/75/58/71 µm. Mean antennal ratio (AR): 0.72 (0.62–0.83; *n* = 25). Mean number 6 and distribution of SCo on flagellomeres 1–13: 2/0/0/0/0/1/0/1.3/1/1/0/0/0; range in number of SCo on flagellomeres 1–13: 2/0/0/0/0/1–2/0/1–2/1/1/0/0/0. Number and distribution of sensilla chaetica on flagellomeres 11–13: 5/4/0. Number and distribution of long and short-tipped sensilla trichodea on flagellomeres 1–10: two long and two short on flagellomeres 1–4 and one long and one short on 5 and 7, one short on 6 and 8, absent on 9 and 10. Palpus (Figure 4b): Mean PR: 0.8 (0.75–0.85), third segment slender with single deep, wide sensory pit. Mean P/H: 0.48 (0.48–0.49).

*Thorax.* Wing (Figure 4a). Mean length: 0.72 mm (0.62 mm – 0.83 mm). Mean costal ratio (CR): 0.46 (0.45–0.48). Mean wing length/width ratio: 2.2 (2.1–2.3; *n* = 25). Legs: middle leg bearing a pair of spines on apices of tarsomeres 1–4.

*Abdomen.* Genitalia (Figure 4c, e, f). Gonocoxite signifi-cantly broader basally, tapering distally; dorsal root long and stout, ventral roots long with anterior processes connected by a very narrow hyaline membrane, posterior heel better developed than the anterior toe (Figure 3f). Paramere (Figure 4e) with a large, anterolaterally directed basal knob; main stem straight, stout, with a large subapical ventral lobe, distal portion recurved to a slender, pointed apex, and with 5–6 lateral fringing spines. Aedeagus (Figure 4f) with a broad, deep basal arch, lateral arms slender, bases curving laterally; shoulders of aedeagus with a pair of posteriorly directed, pointed processes; distal median process long and stout, with truncate apex. Ninth sternum with shallow caudomedian excavation, ventral membrane not spiculate. Ninth tergum with short slender, pointed apicolateral processes (Figure 4c).

## Bionomics

The immature stages, larval habitat, blood-feeding habits and vector status of *C. herero* remain undescribed.

## Distribution

Africa: South Africa: Eastern, Northern and Western Cape provinces. Central and southern Namibia: Erongo, Karas, Khomas and Otjozondjupa regions.

***Culicoides coarctatus*** Clastrier & Wirth 1961 Nigeria

Specimens examined (25 females, 8 males):

- South Africa: Free State: Bethulie (-30.63, 25.88), 4/iii/1970, RS du Plessis 3 females; Limpopo: Groblersdal (-25.14, 29.378), 21/ii/1970, 10 females; Eiland (-23.66, 30.67), 2/iii/1984, R Meiswinkel, 1 male; Thabazimbi: (-24.03, 27.30), 7/xi/1973 A L Dyce 2 females, 1 male; Gauteng: Heuningneskrans (-25.58, 28.19), 23/xi/1978, 6/xi/1979, 7/iv/1984, 3/iii/1988, R Meiswinkel 5 females, 4 males; Onderstepoort (-25.65, 28.19), 7/v/2010, K Labuschagne, 1 female; Mpumalanga Kruger National Park, Mpondo Dam (-25.20, 31.72), 24/ii/1986 R Meiswinkel, 3 females, KwaZulu-Natal Hluhluwe (-28.03, 32.36)10/x/2010 K Labuschagne 1 female, 2 males.

### Differential diagnosis

Medium-sized, fresh specimens dark brown and brown in slide mounted specimens. Female eyes moderately separated. Distal antennal segments not elongated, SCo on each of flagellomeres: 1, 3, 5, (6), 7, 9, 11, 12 in females and 1, 3, 5, 7, 11, 12 in males. Occasionally SCo absent on flagellomere 6 in females and on flagellomeres 3, 5 and 7 in males. The third segment of maxillary palpus moderately expanded, with a large, shallow sensory pit. Wings were patterned with a large pale spot over r-m crossvein extending anteriorly to costal margin and caudally into cell M2; cell r3 with a pale spot on anterior margin just distad of second radial cell; pale spots in distal portion of cells M1, M2, M4, and anal cell; cell M1 with a pale spot near base; cell M2 with a pale streak in basal half and a pale spot in distal portion below basal pale spot in cell M. Spermathecae: Two ovoid spermathecae with third rudimentary spermatheca and sclerotised ring present. Male Genitalia: Ninth tergum with long, basally stout apirolateral processes; ninth sternum with a deep caudomedian emargination, the ventral membrane spiculate; dorsal root of basistyle moderately short and stout, ventral root absent; aedeagus with a shallow basal arch, distal median process short and stout with truncate apex; parameres fused subbasally by a sclerotised bridge, forming a pair of long, slender, bladelike processes with pointed apices.

### Description

**FIGURE 5:**
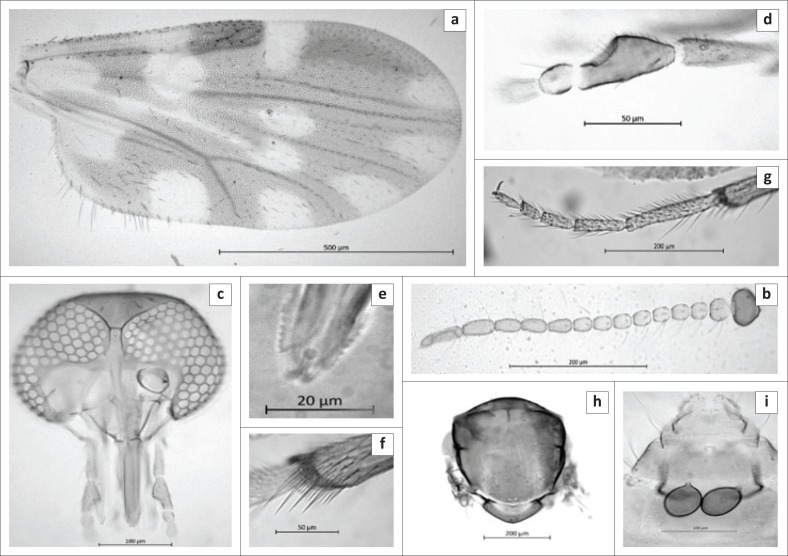
Female of Culicoides coarctatus. (a) wing (female), (b) wing (male), (c) head (female), (d) abdomen showing the spermathecae and the chitinous plates surrounding the gonopore, (e) Male genitalia, (f) Aedeagus. (g) Paramere mandibular teeth, (h) antenna female, (i) antenna male.

Female (Figure 5): *n* = 25.

*Head* (Figure 5c). Eyes bare, narrowly separated by distance of one ocular facet. Flagellum (Figure 5b):

- Lengths of flagellomeres 1–13: 38/28/29/28/30/29/29/30/41/40/46/52/71 µm. Mean antennal ratio (AR) 1.02 (0.98–1.13). Mean number 12 and distribution of SCo on flagellomeres 1–13: 3.2/0.1/1/0.3/1/0.7/1/0.3/1/0.4/1/2.7/0; range in number of SCo on flagellomeres 1–13: 3-5/0-1/1/0-1/1/0-1/1/0-1/1/0-1/1/3-5/0. Number and distribution of sensilla chaetica on flagellomeres 1–13: 6/6/6/6/6/6/5/5/2/2/2/4/5. Number and distribution of long and short blunt-tipped sensilla trichodea on flagellomeres 1–13: two long (LL) on flagellomere 1 and two long (LL) and one short (c) on flagellomeres 2–8. Palpus (Figure 5D) with lengths of segments 1 + 2, 3 - 5: 64/57/29/24. Mean palpal ratio (PR) 2.56 (2.43–2.62); third segment moderately inflated with single, moderately deep and wide sensory pit. Mean P/H ratio 0.85 (0.82–0.87). Mandible (Figure 5E) with 10–13 teeth.

*Thorax* (Figure 5h). Scutum brown in slide mounted material and dark brown in fresh specimens. No distinct pattern could be observed in ethanol preserved or slide mounted material. Halter pale. Wing (Figure 5a):

- Mean length: 0.92 mm (0.88 mm – 98 mm). Mean costal ratio (CR): 0.54 (0.53–0.56). Mean wing length/width ratio: 1.99 (1.90–2.04). Wing patterned, with a large pale spot over r-m crossvein extending anteriorly to costal margin and caudally into cell M2; cell R5 with a pale spot on anterior margin just distad of second radial cell; pale spots in distal portion of cells M1, M2, M4, and anal cell; cell M1 with a pale spot near base; cell M2 with a pale streak in basal half and a pale spot in distal portion below basal pale spot in cell M. Legs (Figure 5g) brown, tarsi paler, knees dark, tibiae with pale basal ring, only middle leg bearing a pair of short, erect spines apically on tarsomeres 1–4. Hind tibial comb (Figure 5f) with four spines with the second spine the longest. Mean tarsal ratio (T/R): 1.72 (1.56–1.80).

*Abdomen*: Two, fully functional, ovoid spermathecae, each with slender, moderately short, narrow neck (Figure 5i) measuring 55.1 µm × 44.5 µm and 51.1 µm × 41.1 µm, and a third rudimentary spermatheca slender measuring 15.6 µm × 4 µm.

**FIGURE 6:**
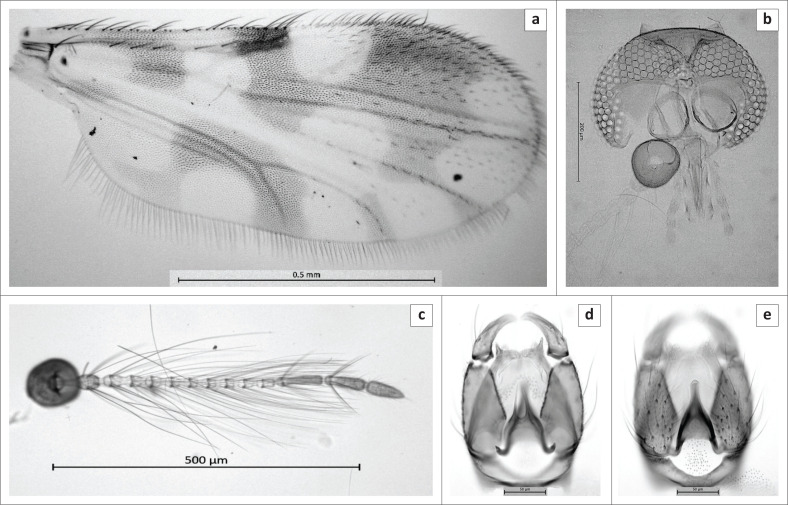
Male of *Culicoides coarctatus* sp. n. (a) Wing, (b) Antenna, (c) Head, (d) Parameres, (e) Aedeagus.

**Male (Figure 6): *n* = 8.**

*Head* (Figure 6b). Eyes bare, separated by less than half width of an ommatidia. Flagellum (Figure 6c): Lengths of flagellomeres 1–13/75/33/29/27/25/25/25/25/25/28/78/72/77 µm:

- Mean antennal ratio (AR): 0.97 (0.88–1.06). Mean number 7 and distribution of SCo on flagellomeres 1–13: 2/0.1/0.7/0.3/0.7/0.3/0.7/0.30/2.3/3/0; range in a number of SCo on flagellomeres 1–13: 2/0–1/0–1/0–1/0–1/0–1/0–1/0–1/0–1/0/1–3/2–3/0. Number and distribution of sensilla chaetica on flagellomeres 11–13: 6/7/0. Number and distribution of long- and short-tipped sensilla trichodea on flagellomeres 1–10: two long and two short on flagellomeres 1–4 and two short on 5–10. Palpus (Figure 6b): Mean PR: 1.98 (1.66–2.27), third segment inflated with single deep, wide sensory pit. Mean P/H: 0.53 (0.48–0.58).

*Thorax.* Scutum brown in colour. Wing (see Figure 6a). Mean length: 0.8 mm (0.68 mm – 0.89 mm). Mean costal ratio (CR): 0.49 (0.46–0.52). Mean wing length/width ratio: 2.3 (2.2–2.4). Legs: middle leg bearing a pair of spines on apices of tarsomeres 1–4.

*Abdomen.* Genitalia (Figure 6d–e) stout, square. Gonocoxite significantly broader basally, ventral root absent; dorsal root stout, moderately long and straight, tip broadly rounded. Gonostyle basal two-thirds broadly inflated, narrowing quite abruptly to sharply pointed tips. Parameres broadly fused in mid-line, the distal portion extending posteriad, each paramere tapering into long, slender, slightly bowed, inwardly curved, fine, erect point (Figure 6e). Aedeagus robust, triangular, well-pigmented, narrowing gradually to broad apex, aedeagal arch moderately high, slightly convex, basal feet well-developed, projecting laterad at right angles (Figure 6d). Sternum 9 with broad and moderately deep caudomedian excavation, posterior membrane rather densely spiculate, spiculation extending posteriad over basal arch of aedeagus (Figure 6d). Tergum 9 trapezoidal, narrowing gradually, apicolateral processes moderately broadly separated each process short, broad basally; caudal margin of tergum with small, indistinct notch medianally.

## Bionomics

The immature stages, larval habitat and vector status of *C. coarctatus* remain undescribed. Blood-feeding habits are unknown although females were collected feeding on humans in Chad (Glick 1990).

## Distribution

Africa: Botswana, Chad, Ethiopia, Kenya, Namibia, Nigeria, South Africa and Zimbabwe.

## Sequencing and phylogenetic analysis

**FIGURE 7:**
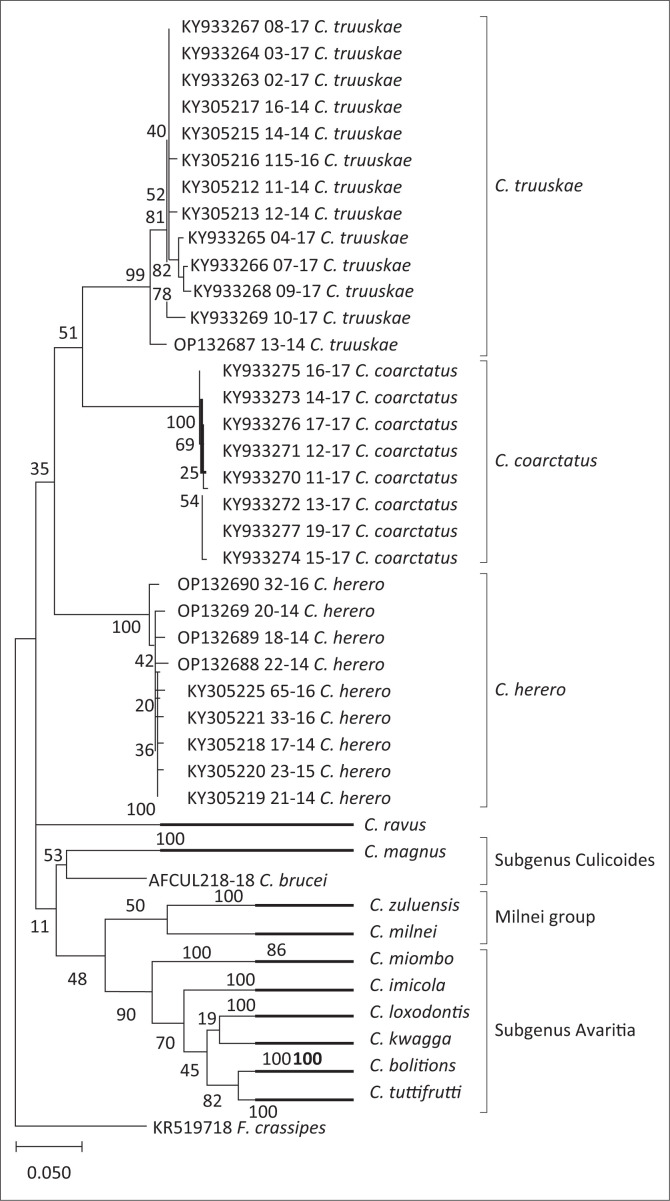
Molecular phylogenetic analysis of *cytochrome c oxidase* I partial gene fragment using maximum likelihood method. The number of bootstrap trees in which the taxa clustered together are indicated next to the branches as a percentage. The tree is to scale, with branch lengths measured in the number of substitutions per site.

The phylogenetic relationship between *C. truuskae* sp. n., with other species were constructed based on the partial COI sequences, with *Forcipomyia crassipes* as an outgroup (Figure 7). Both males and females of *C. truuskae* sp. n., *C. herero* and *C. coarctatus* were sequenced. The phylogenetic analysis of all three species clearly indicate that the males and females belong to the same species, with more than 97% sequence similarity within the three groups. An average of 88% sequence similarity was observed between the sequences of *C. truuskae* sp. n. and either *C. herero* or *C. coarctatus* species, while all the other sequences shared less than 84% sequence similarity (Table 1). Sequences from 11 additional species were obtained from Genbank and included into the phylogenetic analysis. The related species clustered together in three distinct clades, that is subgenera *Avaritia, Culicoides* as well as the Milnei group. The clustering of *C. truuskae* sp. n. and *C. coarctatus* together is weakly supported with a bootstrap value of 51, and both species cluster weakly with *C. herero* with a bootstrap value of 35. In the mean genetic distances, *C. truuskae* sp. n. share the highest percentage sequence identity with *C. herero* and *C. coarctatus* compared with the other species (Table 1). In order to establish the relatedness of these three species, additional nuclear gene regions will need to be sequenced.

**TABLE 1:** Mean genetic distances of the *cytochrome c oxidase* I region between and within species.

Species	Mean interspecific distance			Mean intraspecific distance	
	1	2	3	Species	Intraspecific distance
1	-	-	-	*C. truuskae*	0.013
2	0.127	-	-	*C. coarctatus*	0.003
3	0.129	0.160	-	*C. herero*	0.008
4	0.160	0.164	0.185	*C. ravus*	0.004
5	0.171	0.170	0.205	*C. magnus*	0.000
6	0.171	0.170	0.166	*C. brucei*	0.000
7	0.241	0.222	0.242	*C. zuluensis*	0.004
8	0.208	0.224	0.215	*C. milnei*	0.010
9	0.187	0.170	0.184	*C. miombo*	0.000
10	0.195	0.180	0.177	*C. imicola*	0.006
11	0.197	0.221	0.213	*C. loxodontis*	0.000
12	0.200	0.192	0.200	*C. kwagga*	0.000
13	0.207	0.200	0.207	*C. bolitinos*	0.000
14	0.178	0.182	0.177	*C. tuttifrutti*	0.005

Note: 1: *Culicoides truuskae,* 2: *Culicoides coarctatus,* 3: *Culicoides herero,* 4: *Culicoides ravus,* 5: *Culicoides magnus,* 6: *Culicoides brucei,* 7: *Culicoides zuluensis,* 8: *Culicoides milnei*, 9: *Culicoides miombo,* 10: *Culicoides imicola,* 11: *Culicoides loxodontis,* 12: *Culicoides kwagga,* 13: *Culicoides bolitinos,* 14: *Culicoides tutti-frutti.*

## Discussion and conclusion

In *C. truuskae* sp. n., the charcoal-coloured smudge broadly running the length of cell r3 is diagnostic and are similar to the one seen in the wing of *C. herero.* In his original description of *C. herero,* based on a female from Namibia, Enderlein (1908) singled out this smudge and, in German, referred to it as a ‘*verwaschene Fleck*’ (*vF*). Translated, this refers to something as ‘washed out, faded, watery, blurred’; these nuanced meanings aptly capture the nature of the ‘*Fleck*’ and, although obvious, its margins are indistinct rather than sharply defined. The *vF* is found on the wing in both *C. herero* and *C. truuskae* sp. n. and because these two species occur in sympatry, raises the point as to which species Enderlein was dealing with when he briefly described *C. herero* over a century ago. Although long considered ‘lost’, the discovery of the holotype in the holdings of the Berlin Museum enabled Szadziewski (1986) to re-examine it; along with a full description, he also provided a figure of the wing clearly showing the *vF* as well as the first and second costal spots. In contrast with the single female that Szadziewski (1986) examined, the majority of our specimens had a very indistinct pale spot in cell cua1. Unfortunately, the Namibian samples available were collected 250 km from Rooibank where the holotype was collected. As to its taxonomic position, Szadziewski concluded, correctly, that *C. herero* is a member of the Similis species group (one of the many clades that comprise the subgenus *Oecacta* Poey). Szadziewski’s full redescription of the holotype of *C. herero* makes it clear that Enderlein did not have *C. truuskae* sp. n. before him all those years ago (Szadziewski 1986).

After a 2-year weekly light-trap survey conducted over 30 months (between 1996 and 1999) at 40 livestock holdings distributed across South Africa, *C. coarctatus* was found at 21, *C. herero* at 17 and *C. truuskae* sp. n at 9 of the sites. Monthly abundance data for *C. coarctatus* was only available for 18 sites, *C. herero* for 8 sites and *C. truuskae* sp. n for 7 sites.

**FIGURE 8:**
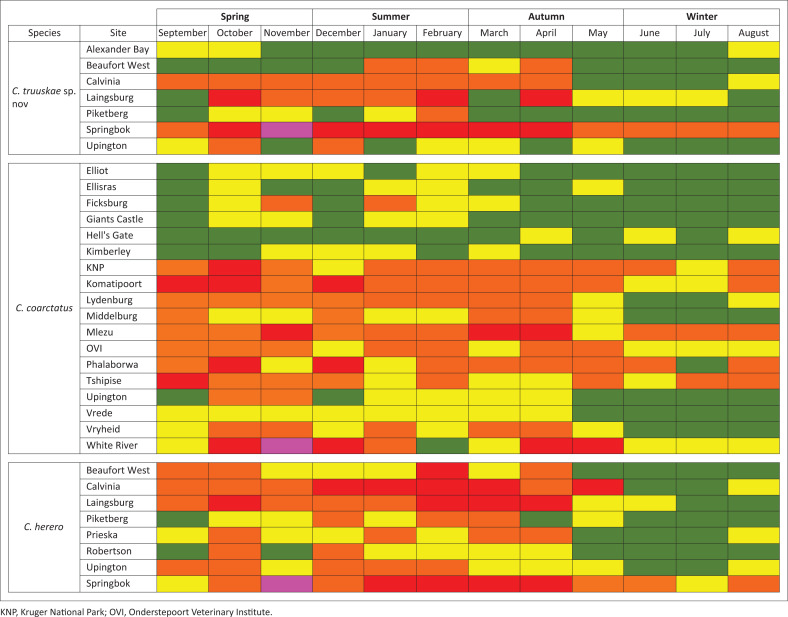
Mondrian matrix of the monthly abundances for *Culicoides truuskae* sp. n., *Culicoides coarctatus* and *Culicoides herero* (colour coded); *Culicoides* – green, 0; yellow, 1–9; orange, 10–99; red, 100–999; pink, 1000–9999.

To highlight similarities and differences between *C truuskae* sp. n., *C. coarctatus* and *C. herero*, the monthly prevalence data are presented in the Mondrian matrix (Figure 8); to facilitate their comparison, the relative weekly abundances are colour coded (Figure 8 – legend). All three species peaked in September with monthly averages above 1000 specimens. The largest collection of *C. truuskae* sp. n. comes from the farm Koperberg in the Springbok area in the Northern Cape Province of South Africa where it comprised 1 421 (12.29%) of a total of 11 564 *Culicoides* collected. The largest collection of *C. herero* comes from the farm Aus in Namibia where it comprised 3108 (53.51%) of a total of 5808 *Culicoides* collected. The largest collection of *C. coarctatus* comes from the farm Lisbon in the Hazyview area in Mpumalanga, South Africa where it comprised 1002 (0.34%) of a total of 293 497 *Culicoides* collected.

**FIGURE 9:**
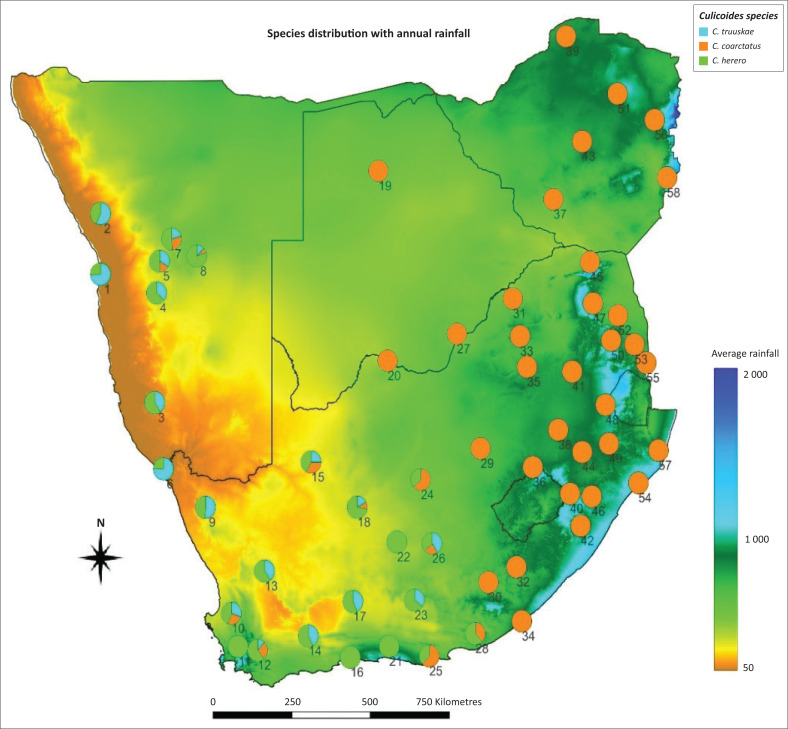
Average rainfall and distribution map of *Culicoides truuskae* sp. n., *Culicoides coarctatus* and *Culicoides herero* in Southern Africa. Pie charts symbols depict the proportional distribution of each species per collection area.

The respective geographic ranges of *C. truuskae* sp. n. and *C. coarctatus* verge on allopatry (Figure 9). Since its description almost 60 years ago, the seasonal phenology of *C. coarctatus* has remained ‘… virtually unknown’, in spite of its wide occurrence within the savannas of Africa, from Nigeria and Chad to Ethiopia in the north, down to Kenya, Zimbabwe and South Africa in the south (Glick 1990). All the sites for *C. coarctatus* were located in the eastern two-thirds of South Africa. Although uncommon and confined broadly to areas experiencing an annual average rainfall in excess of 400 mm, *C. coarctatus* abundance peaked at sites that lay within the hotter, drier, frost-free subtropical grasslands, savannas and shrublands that comprise ecoregion 54 (eds. Mucina & Rutherford 2006) and which are situated along the eastern margin of the country. For example, at the frost-free sites of Komatipoort, Kruger National Park, Phalaborwa, Tshipise White River and Mlezu (Zimbabwe), *C. coarctatus* occurred in light traps throughout the year, peaked twice, once in abundance from September to December and again from March to May. These months are extremely hot and from September to December it is also extremely dry when the summer rains hold off. The abundances in these hot and dry months could be because of the collection site choice. To ensure that large numbers of *Culicoides* are collected, light traps are operated near livestock at livestock holdings, that were kept close to watering points, where water spillage occurred through leaking troughs.

*Culicoides coarctatus* and *C. truuskae* sp. n. have near allopatric ranges while *C. herero* and *C. truuskae* sp. n. have sympatric ranges. In sharp contrast to *C. coarctatus,* the geographical range of *C. truuskae* sp. n. is restricted to the northern, western and eastern Cape provinces, but this species is seldom found abundantly in light trap collections (Figure 9, Online Appendix 1 Table 2-A1). *Culicoides truuskae* sp. n. and *C. herero* occur in the Fynbos, Nama-Karoo and Succulent Karoo ecoregions in South Africa, although *C. herero* also occur in the southern savanna ecoregion of the eastern and western Cape provinces and both occur in the desert and savanna ecoregions in Namibia. Little is known about *C. truuskae* sp. n. abundance, prevalence and seasonal distribution in Namibia owing to limited sampling studies. *Culicoides coarctatus* distribution is wider in all ecoregions except the Succulent Karoo and Desert ecoregions into areas with up to 1500 mm rainfall annually. *Culicoides herero* has a wider distribution than *C. truuskae* sp. n. and penetrates into areas with up to 1000 mm rainfall annually (Figure 9, Online Appendix 1 Table 2-A1).

*Culicoides truuskae* sp. n. and *C. coarctatus* can be distinguished based on their size, wing pattern and SCo distribution on the flagellomeres (Table 2). *Culicoides truuskae* sp. n. is a medium sized species, lacking wing maculation pattern, with SCo on flagellomeres 1, 3, 5, 7, 9–12, while *C. coarctatus* is a much smaller species, with a definite wing maculation pattern (pale spots at apex of the wing margin, with two rows of spots from the first and the second costal spots to the posterior wing margin) and SCo on flagellomeres 1, 3, 5, (6), 9, 11–12. The male genitalia of *C. truuskae* sp. n. and *C. coarctatus* are similar, that is fused parameres and the densely spiculate posterior membrane of sternum 9, with exception of the aedeagal arch that is high and slightly rounded in *C. truuskae* sp. n., but only moderately high and rounded in *C. coarctatus.*

**TABLE 2:** Differential characters for females of *C. truuskae* sp. n., *C. coarctatus* and *C. herero*, with minimum and maximum values in parenthesis.

Intraspecies variation in some numerical characters of female specimens	*C. truuskae* sp. n.	*C. coarctatus*	*C. herero*
Wing length (mm)	1.39 (1.11–1.60)	0.94 (0.87–1.04)	0.88 (0.77–1.05)
Costal ratio (CR)	0.61 (0.59–0.62)	0.56 (0.55–0.57)	0.53 (0.52–0.55)
Antennal ratio (AR)	1.08 (1.06–1.09)	1.02 (0.98–1.13)	0.87 (0.85–0.89)
Sensilla coeloconica	1, 3, 5, 7, 9–12	1, 3, 5–7, 9, 11, 12	1–8
Palpal ratio (PR)	2.02 (1.95–2.05)	2.56 (2.43–2.62)	2.01 (1.92–2.10)
Proboscis/head ratio (P/H)	0.81 (0.79–0.83)	0.85 (0.82–0.87)	0.60 (0.57–0.62)

C. truuskae sp. n., Culicoides truuskae sp. n.; C. coarctatus, Culicoides coarctatus; C. herero, Culicoides herero.

*Culicoides herero* can be distinguished from *C. truuskae* sp. n. and *C. coarctatus* based on size, wing pattern and SCo distribution on the flagellomeres (Table 2). *Culicoides herero* is a small species lacking wing maculation pattern except for very indistinct pale spot in cell cua1 and SCo on flagellomeres 1–8. The aedeagus and parameres of *C. herero* differ from both *C. truuskae* sp. n. and *C. coarctatus.* In *C. herero* the shoulders of aedeagus have a pair of posteriorly directed and the parameres has a large, anterolaterally directed basal knob; main stem straight, stout, with a large subapical ventral lobe, distal portion recurved to a slender, pointed apex and with 5–6 lateral fringing spines.

*Culicoides confusus* Carter, Ingram and Macfie, resemble *C. coartcatus,* in wing pattern, but differ in having SCo on flagellomeres 1, 3, 5–7, 9–12 and a mean lower palpal ratio of 1.93 versus 2.31 in *C. coarctatus.* The males of *C. confusus* have not officially been described, although Boorman and Dipeolu (1979) give a description of the male based on a single specimen. This male differs from *C. coarctatus* in the posterior membrane of sternum 9 being bare and that the parameres end in curved tips. In wing pattern *C. clarkei* Carter, Ingram and Macfie, also resemble *C. coartcatus* but differ in having SCo distribution on the flagellomeres 1–7, 9–12. The male genitalia also differ in having separated parameres and a triangular aedeagus with a long finger-like median process. These two species are currently not known to occur in South Africa.

In conclusion morphology, sequence results based on the phylogenetic analysis of the partial COI gene region support *C. truuskae* sp. n as a valid new species and the description of the male of *C. herero* for the first time.
